# Association Between Dietary Flavonoids Intake and Cognitive Function in an Italian Cohort

**DOI:** 10.3390/biom10091300

**Published:** 2020-09-09

**Authors:** Justyna Godos, Filippo Caraci, Sabrina Castellano, Walter Currenti, Fabio Galvano, Raffaele Ferri, Giuseppe Grosso

**Affiliations:** 1 Oasi Research Institute—IRCCS, 94018 Troina, Italy; justyna.godos@gmail.com (J.G.); carafil@hotmail.com (F.C.); rferri@oasi.en.it (R.F.); 2 Department of Drug Sciences, University of Catania, 95125 Catania, Italy; 3 Department of Educational Sciences, University of Catania, 95124 Catania, Italy; sabrina.castellano@unict.it; 4 Department of Biomedical and Biotechnological Sciences, University of Catania, 95123 Catania, Italy; currentiw@gmail.com (W.C.); fgalvano@unict.it (F.G.)

**Keywords:** flavonoids, anthocyanins, polyphenols, antioxidants, brain, cognitive, neurodegenerative, cohort, population, Sicily

## Abstract

Background: Diet is one of the leading factors contributing to the prevalence of non-communicable diseases, including neurodegenerative disorders. Dietary polyphenols, antioxidant components and anti-inflammatory agents of plant-based foods rich diets have been shown to modulate neuro-inflammation, adult neurogenesis and brain signaling, all of which are linked to cognitive function. As epidemiological evidence is limited and the results are contradictory, the aim of this study is to explore the association between dietary flavonoid intake and cognitive health among the adult population living in the Mediterranean area. Methods: The demographic and dietary habits of 808 adults living in southern Italy were analyzed. Food frequency questionnaires (FFQs) were used to assess dietary intake. Data on the polyphenol content in foods were estimated using the Phenol-Explorer database. The Short Portable Mental Status Questionnaire was used as a screening tool for cognitive status. Multivariate logistic regression analyses were used to assess the associations. Results: A significant inverse association between higher dietary intake of total flavonoids (Q4 vs. Q1: OR = 0.39, 95% CI: 0.15, 1.00) and impaired cognitive status was found. Among individual subclasses of flavonoids, flavan-3-ols (Q3 vs. Q1: OR = 0.30, 95% CI: 0.11, 0.76), catechins (Q4 vs. Q1: OR = 0.24, 95% CI: 0.08, 0.72), anthocyanins (Q4 vs. Q1: OR = 0.38, 95% CI: 0.14, 1.00) and flavonols (Q3 vs. Q1: OR = 0.30, 95% CI: 0.11, 0.76) were associated with cognitive health. Among individual polyphenols, only quercetin was associated with cognitive health (Q4 vs. Q1: OR = 0.30, 95% CI: 0.10, 0.91). Conclusions: The results of this study suggest that higher dietary intake of flavonoids may be associated with better cognitive health among adult individuals.

## 1. Introduction

In recent years, a significant rise in the prevalence of neurodegenerative diseases, including cognitive decline and dementia has been observed [1]. Among modifiable risk factors, dietary factors have been identified as playing a potential role in preserving and possibly improving mental health and cognitive function in older adults [2]. A recent summary of evidence demonstrated the beneficial effect of plant-based foods and dietary patterns toward cognitive health, including fruits and vegetables [3] and higher adherence to the Mediterranean diet [4]. Moreover, consumption of plant-derived beverages, such as tea and coffee, have demonstrated potential favorable effects toward cognitive health and decreased risk of cognitive decline and mood disorders in prospective cohort studies [5,6,7,8].

A common characteristic of dietary patterns and individual foods investigated is their richness in polyphenols, a wide range of molecules that naturally occur in plant foods: these compounds have demonstrated antioxidant and anti-inflammatory properties, which have been hypothesized to play a role in a number of chronic non-communicable diseases, including cancer [9] and cardiovascular diseases [10], while their consumption has been related with a longer lifespan [11]. Experimental studies also demonstrated that flavonoids exert a neuroprotective action through regulation of neuro-inflammation, adult neurogenesis and brain signaling, all of which are linked to cognitive function [12]. However, the evidence on the role of flavonoids in the prevention of neurodegenerative disorders is still limited. The aim of this study was to explore the association between dietary flavonoids intake, their classes as well as individual compounds and cognitive health in an Italian cohort.

## 2. Materials and Methods

### 2.1. Study Population

The MEAL study is an observational study aiming to investigate the association between nutritional and lifestyle habits characterizing the classical Mediterranean area and non-communicable diseases. The baseline data comprised a sample of 2044 men and women aged 18 or more years old. Details of the study protocol are published elsewhere [13]. Briefly, individuals were randomly selected in the main districts of the city of Catania, southern Italy. The enrolment and data collection were performed between 2014 and 2015 through the registered records of local general practitioners stratified by sex and 10-year age groups. The theoretical sample size was set at 1500 individuals to provide a specific relative precision of 5% (Type I error, 0.05; Type II error, 0.10), taking into account an anticipated 70% participation rate. Out of 2,405 individuals invited, the final sample size was 2044 participants (response rate of 85%). Given the outcome investigated has major impact at older ages, the analysis for the present study was restricted to individuals of age of 50 years old or older (n = 916). All participants were informed about the aims of the study and provided a written informed consent. All the study procedures were carried out in accordance with the Declaration of Helsinki (1989) of the World Medical Association. The study protocol has been reviewed and approved by the concerning ethical committee (protocol number: 802/23 December 2014).

### 2.2. Data Collection

An electronic data collection was performed by face-to-face assisted personal interview, using tablet computers. In order to visualize the response options, participants were provided with a paper copy of the questionnaire. However, final answers were registered directly by the interviewer. The demographic data included gender, age at recruitment, highest educational degree achieved, occupation (specifies the character of the most important employment during the year before the investigation) or last occupation before retirement and marital status. Occupational status was categorized as (i) unemployed, (ii) low (unskilled workers), (iii) medium (partially skilled workers), and (iv) high (skilled workers). Educational status was categorized as (i) low (primary/secondary), (ii) medium (high school) and (iii) high (university). Physical activity was evaluated with the International Physical Activity Questionnaires (IPAQ) [14], which included a set of questionnaires (5 domains) investigating the time spent being physically active in the last 7 days. Based on the IPAQ guidelines, the final score allowed us to categorize physical activity level as (i) low, (ii) moderate and (iii) high. Smoking status was categorized as (i) non-smoker, (ii) ex-smoker and (iii) current smoker, while alcohol consumption was categorized as (i) none, (ii) moderate drinker (0.1–12 g/d) and (iii) regular drinker (>12 g/d). Health status included information about anthropometric measurements assessed through standard methods and previous or current cardiometabolic diseases and cancer [15].

### 2.3. Dietary Assessment

The dietary assessment was performed by the administration of two food frequency questionnaires (FFQ, a long and a short version) that have been previously tested for validity and reliability for the Sicilian population [16,17]. The identification of the food intake, the energy content as well as the macro- and micro-nutrients intake were obtained through comparison with food composition tables of the Italian Research Center for Foods and Nutrition [18]. Intake of seasonal foods referred to consumption during the period in which the food was available and then adjusted by its proportional intake in one year. FFQs with unreliable intakes (<1000 or >6000 kcal/d) were excluded from the analyses (n = 33) leaving a total of 883 individuals included in the analysis.

### 2.4. Dietary Flavonoid Intake Estimation

The process of the estimation of habitual flavonoid intake has been previously described in detail [19]. Briefly, data on the flavonoid content in foods were retrieved from the Phenol-Explorer database [20]. First, all foods that contained no polyphenols were excluded from the calculation, leaving a total of 75 food and beverage items included for the polyphenol estimation (including mostly fruits, vegetables, cocoa, tea and coffee, but also olive oil, nuts and alcoholic beverages). The food consumption was calculated (in g or ml) by following the standard portion sizes used in the study and then converted in 24-h intake; then, the Phenol-Explorer database was searched to retrieve mean content values for all flavonoids contained in the foods obtained and flavonoid intake from each food was calculated by multiplying the content in total, main subclasses, and selected individual flavonoids by the daily consumption of each food adjusted for total energy intake (kcal/d) using the residual method [21].

### 2.5. Cognitive Evaluation

Cognitive health was evaluated using the Short Portable Mental Status Questionnaire (SPMSQ), a 10-item tool administered by the clinician in the office or in a hospital [22]. The pre-defined categories for interpretation of the screening tool were (i) intact status, less than 3 errors; (ii) mild impaired, 3 to 4 errors; (iii) moderate impaired, 5 to 7 errors, and (iv) severe impaired status, 8 or more errors. For this study, we considered severe impaired status as cut off point for impaired cognitive health.

### 2.6. Statistical Analysis

Frequencies were expressed as absolute numbers and percentages, while continuous variables were expressed as means and standard deviations. Individuals were grouped based on variables of exposure (dietary flavonoid intake) and distribution of background characteristics were compared between groups. Differences were tested with the Chi-square test for categorical variables, ANOVA for continuous variables distributed normally and the Kruskall–Wallis test for variables distributed not normally. Energy-adjusted multivariate logistic regression models were used to test the association between dietary flavonoid intake (total, subclasses and individual flavonoids) and cognitive status. A multivariate model adjusted for all other background characteristics (sex, age, BMI, physical activity, educational status, occupational status, smoking status, alcohol consumption, occurrence of hypertension, diabetes, dyslipidaemias, cardiovascular disease, cancer, menopausal status) was also performed in order to test whether the observed associations were independent from the aforementioned variables. All reported P-values were based on two-sided tests, and the significance level was 5%. SPSS 17 (SPSS Inc., Chicago, IL, USA) software was used for all the statistical analyses.

## 3. Results

### 3.1. Background Characteristics

The main baseline demographic, socio-economic and health characteristics of the cohort distributed by the quartiles of energy-adjusted total polyphenol intake are presented in Table 1. Individuals in the third and fourth quartile of total polyphenol intake were significantly younger, and there were more men in the first and third quartile of exposure. Similarly, significant differences were observed in the distribution of educational level, yet with no linear trend. Participants in the highest quartile of total polyphenol intake were moderate or regular alcohol drinkers. No significant differences among quartiles of total polyphenol intake were found when considering health status, including prevalence of hypertension, type-2 diabetes, dyslipidaemias, cardiovascular diseases and cancer (Table 1). Higher total polyphenol intake was also in line with higher total energy intake (Table 1).

### 3.2. Flavonoid Intake

Mean intake and standard deviation of total, subclasses and individual flavonoid by cognitive status are presented in Table 2. A significant difference was found solely in the dietary intake of anthocyanins between individuals with normal and impaired cognitive function (57.03 ± 54.79 mg/d vs. 42.14 ± 44.18 mg/d, respectively).

### 3.3. Association Between Flavonoid Intake and Cognitive Health

Multivariate adjusted analysis revealed a significant association between total dietary flavonoid and cognitive status; however, no clear linear trend was observed (Table 3). Among individual subclasses of flavonoids, individuals in the highest quartile of catechin and anthocyanins intake were less likely to have impaired cognitive status (OR = 0.38, 95% CI: 0.14, 1.00), while when considering flavan-3-ols (OR = 0.30, 95% CI: 0.11, 0.76) and flavonols (OR = 0.30, 95% CI: 0.11, 0.76) only individuals in the third quartile of intake were less likely to have impaired cognitive status. Conversely, higher dietary intake of flavanones (OR = l.05, 95% CI: 0.43, 2.55; Table 3) and flavones (OR = 0.73, 95% CI: 0.31, 1.69; Table 3) was not associated with better cognitive status, independently of the covariates considered in the analysis (Table 3). When considering the highest quartile of intake, similar results were observed for the individual flavonoids belonging to flavanones and flavones subclasses, including hesperetin (OR = 1.20, 95% CI: 0.49, 2.92) and naringenin (OR = 1.00, 95% CI: 0.41, 2.39) as well as apigenin (OR = 0.92, 95% CI: 0.40, 2.12) and luteolin (OR = 0.90, 95% CI: 0.40, 2.00), respectively (Table 3). When considering individual polyphenols, only quercetin was significantly associated with cognitive health, as participants in the highest quartile of intake were less likely to have impaired cognitive status (OR = 0.30, 95% CI: 0.10, 0.91; Table 3).

A stratified analysis by sex showed an association between impaired cognitive health and total flavonoids in men and women (for to the third vs. the first quartile of intake, OR = 0.08, 95% CI: 0.02, 0.39 and OR = 0.21, 95% CI: 0.09, 0.52), flavonols in men (for to the third vs. the first quartile of intake, OR = 0.17, 95% CI: 0.03, 0.86), apigenin in women (for to the third vs. the first quartile of intake, OR = 0.33, 95% CI: 0.12, 0.88) and catechins in women (for to the forth vs. the first quartile of intake, OR = 0.24, 95% CI: 0.08, 0.75), with no other significant results (data not shown).

A subgroup analysis by age showed an association between impaired cognitive health and total flavonoids (for to the third vs. the first quartile of intake, OR = 0.12, 95% CI: 0.04, 0.33), flavanones (for to the forth vs. the first quartile of intake, OR = 0.06, 95% CI: 0.01, 0.47), hesperetin (for to the forth vs. the first quartile of intake, OR = 0.06, 95% CI: 0.01, 0.51) and naringenin (for to the forth vs. the first quartile of intake, OR = 0.19, 95% CI: 0.06, 0.63) in individuals over 65 years old, with no other significant results (data not shown).

## 4. Discussion

The results of the present study showed that higher dietary intake of flavonoid and certain subclasses was associated with better cognitive health among Italian adults. In particular, higher dietary intake of flavan-3-ols, catechins, flavonols, anthocyanins and, among individual molecules, quercetin was positively associated with cognitive status. Total flavonoids were associated with better cognitive health in both men and women, with small differences between sexes; concerning elderly individuals, flavanones and the individual compounds investigated hesperetin and naringenin were also associated with cognitive health.

Dietary flavonoids have been linked to several mental health outcomes, including depression [23,24] and sleep disorders [25], while other studies showed an association with a variety of cognitive health outcomes. Regarding flavonoid intake, data from Nurses’ Health Study remarked our results showing that total flavonoid intake was associated with slower rates of cognitive decline in 16,010 participants, aged ≥70 years [26]. Furthermore, in the PAQUID (*Personnes Agées Quid*) study, a cohort including 1640 subjects aged 65 years or older free from dementia at the baseline interview, flavonoid intake was associated with better cognitive performance at baseline and with a better evolution of the performance over 10-year follow-up [27].

We also found an association between specific flavonoid subclasses in line with previous reports from the scientific literature. The SU.VI.MAX. (*Supplementation en Vitamines et Mineraux Antioxydants*) study included 2,574 middle-aged adults followed for 13 years and found that among specific groups of flavonoids, both catechins and flavonols resulted in an association with better cognitive function, in line with our results [28]. Furthermore, in another study conducted on a large cohort of about 10,000 individuals aged 45–64 flavonol intake has been shown to be positively associated with preserved combined cognitive function; the result was consistent for the three major individual flavonols in the diet, i.e., myricetin, kaempferol, and quercetin [29]. In contrast, in another study conducted on the Framingham Offspring cohort, long-term dietary flavonoid intake was associated with slower cognitive decline, with independent association of higher flavan-3-ol intake, the other subgroup of flavonoids resulting associated with cognitive health in our study [30].

Among trials investigating dietary interventions to affect cognitive decline and function, the general results are confirming those reported in our study. A study conducted in the frame of the PREDIMED (*Prevención con Dieta Mediterránea*) study reported that urinary polyphenols (measured as markers of dietary polyphenol consumption) were associated with better scores in immediate verbal memory [31]. Regarding the classes of flavonoids potentially involved, urinary concentrations of specific flavan-3-ol metabolites (from a polyphenol-rich extract of grape and blueberry) were associated, at the end of a 6-month intervention study conducted on 215 elderly subjects (60–70 years-old), with memory improvements and a better verbal episodic and recognition memory-delayed recognition [32]. These findings are in line with the results of another intervention study conducted in 90 elderly individuals without clinical evidence of cognitive dysfunction: in this case, (cocoa) flavan-3-ols were shown to reduce outcomes of age-related cognitive dysfunction [33].

Several mechanisms have been hypothesized to support the effect of dietary flavonoids toward cognitive health, including both direct and indirect pathways of action. Flavonoids may indirectly affect brain health by modulating systemic inflammation and oxidative stress, key risk factors related to aging and playing a crucial role in the pathogenesis of neurodegenerative disorders [34]. In fact, flavonoids have been hypothesized to affect production, bioavailability and biological activity of metabolites linked to the gut-microbiome-brain axis [35]. Gut microbiota imbalance is associated with local and systemic inflammatory status, which in turn may affect immune and nervous system-related diseases [36]. Specifically, higher inflammatory status triggers vascular damage and neuro-inflammation, which in turn may cause disruptions in brain structure and function, including ion homeostasis, regulation of metabolic functions, production of anti-oxidant species, levels of synaptic glutamate, modulation of synaptic plasticity, and finally maintenance of the brain–blood barrier (BBB) [37]. These mechanisms are supported by some clinical studies, which demonstrated a significant association between systemic inflammation and oxidative stress and cognitive decline [38,39].

Recently, the direct mechanisms through which flavonoids could exert neuroprotective effects have gained great attention mainly due to the discovery that certain flavonoid metabolites pass the BBB and have a direct impact on neuronal cells [40,41]. Among the compounds found to be associated with better cognitive health in our study, flavonols and flavanones have been shown to increase the transforming growth factor-β1 (TGF-β1) levels [42,43], a neurotrophic factor that exerts a key role in recognition memory formation, and a deficit of TGF-b1 signaling can contribute to cognitive decline in Alzheimer’s disease [44,45]. With special regard to quercetin (a flavonol found related to better cognitive health in our study), it has demonstrated effectiveness in animal models of Alzheimer’s disease in decreasing of oxidative stress [46] and positively modulating Nrf2/HO-1 (nuclear factor erythroid 2–related factor 2/heme oxygenase-1) pathway [47]; also, an effect toward neuro-inflammation through regulation of NF-κB (nuclear factor kappa-light-chain-enhancer of activated B cells) and has been observed [48]. Furthermore, quercetin has been shown to induce the expression of a brain-derived neurotrophic factor (BDNF) [49] as well as a nerve growth factor (NGF) [50], which are the most studied and best-characterized neurotrophins of the central nervous system, involved in the modulation of synaptic plasticity and adult neurogenesis. In line, similar results have been reported for catechins [51] and anthocyanins [52], other subgroups of flavonoids reported to be associated with better cognitive health in our study. The role of flavonoid-rich foods in improving cognitive health linked to changes in serum BDNF levels has also been reported in the clinical setting [53]. Thus, future studies may consider studying flavonoids able to modulate TGF-β1 release, BDNF and NGF as new pharmacological tools to improve cognitive function.

The findings of the present study should be considered in light of some limitations. First, this study provided evidence from a cross-sectional analysis, and thus a causal relationship cannot be established. Second, all methods used to assess food consumption and dietary polyphenol intake, even though commonly used and valid as tools applied in nutrition epidemiology, are subjected to recall bias and provide only an estimation, while the real intake and especially exposure to metabolites cannot be estimated. Third, this study explored the association based on information of dietary flavonoid intake and thus the inter-individual variation in response to consumption of flavonoid and actual exposure to the flavonoid metabolites could not be taken into account. Finally, although validated, the SPMSQ may still suffer from limitations in sensitivity and specificity, especially for individuals with limited education.

## 5. Conclusions

The findings of the present study suggest that higher dietary intake of flavonoids and their subclasses, in particular anthocyanins, flavan-3-ols, catechins and flavonols are related to cognitive health. Further studies are needed to better elucidate the nature of the association between habitual dietary intake of flavonoids and cognition in order to assess whether a protective effect might explain the retrieved findings. Based on the results of this study, future research should focus on selected flavonoids and also emphasize the inter-individual variation in response to consumption of polyphenols, thus investigating the association not only with their dietary intake but also their metabolites. As a future perspective, a focus on the gut microbiota composition should be considered as differences in microbial flora may impact polyphenol metabolite formation and bioactivity.

## Figures and Tables

**Table 1:** Background characteristics of study participants by quartiles of total polyphenol intake (energy-adjusted).

	Total Polyphenol Intake				*P*
	Q1 (n = 184)	Q2 (n = 237)	Q3 (n = 253)	Q4 (n = 209)	
Age (years), mean (SD)	67.3 (11.1)	65.2 (9.2)	63.9 (8.9)	63.5 (8.6)	<0.001 ^a^
Men, n (%)	124 (67.4)	124 (52.3)	146 (57.7)	107 (51.2)	0.004 ^b^
BMI, mean (SD)	26.5 (4.4)	26.7 (4.3)	27.04 (4.4)	27.2 (3.9)	0.414 ^a^
Smoking status, n (%)					0.665 ^b^
Current	43 (23.4)	56 (23.6)	53 (20.9)	47 (22.5)	
Former	30 (16.3)	52 (21.9)	56 (22.1)	49 (23.4)	
Never	111 (60.3)	129 (54.4)	144 (56.9)	113 (54.1)	
Educational level, n (%)					0.003 ^b^
Low	105 (57.1)	120 (50.6)	114 (45.1)	112 (53.6)	
Medium	58 (31.5)	71 (30.0)	103 (40.7)	53 (25.4)	
High	21 (11.4)	46 (19.4)	36 (14.2)	44 (21.1)	
Occupational level, n (%)					0.086 ^b^
Unemployed	47 (30.3)	45 (21.6)	54 (23.8)	57 (31.1)	
Low	31 (20.0)	43 (20.7)	35 (15.4)	26 (14.2)	
Medium	33 (21.3)	69 (33.2)	79 (34.8)	53 (29.0)	
High	44 (28.4)	51 (24.5)	59 (26.0)	47 (25.7)	
Physical activity level, n (%)					0.169 ^b^
Low	52 (33.8)	55 (27.0)	46 (21.7)	43 (24.0)	
Medium	63 (40.9)	100 (49.0)	116 (54.7)	91 (50.8)	
High	39 (25.3)	49 (24.0)	50 (23.6)	45 (25.1)	
Alcohol consumption, n (%)					<0.001 ^b^
None	58 (31.5)	62 (26.2)	41 (16.2)	29 (13.9)	
Moderate (0.1–12 g/d)	121 (65.8)	152 (64.1)	151 (59.7)	91 (43.5)	
Regular (>12 g/d)	5 (2.7)	23 (9.7)	61 (24.1)	89 (42.6)	
Health status, n (%)					
Hypertension	145 (78.8)	185 (78.1)	184 (72.7)	146 (69.9)	0.103 ^b^
Diabetes	21 (11.4)	50 (21.1)	41 (16.2)	32 (15.3)	0.061 ^b^
Dyslipidaemias	59 (32.1)	89 (37.6)	90 (35.6)	64 (30.6)	0.398 ^b^
Cardiovascular disease	37 (20.6)	32 (13.9)	37 (15.2)	30 (15.1)	0.285 ^b^
Cancer	17 (9.2)	17 (7.2)	18 (7.1)	22 (10.5)	0.492 ^b^
Menopausal status (women only), n (%)	11 (8.6)	13 (10.2)	18 (11.8)	6 (5.3)	0.320 ^b^
Total energy intake (kcal/d), mean (SD)	1768.3 (534.1)	1900.1 (512.1)	2026.9 (559.1)	2486.1 (765.9)	<0.001 ^a^

^a^ Differences were assessed by ANOVA test. ^b^ Differences were assessed by Chi-Square test.

**Table 2:** Mean intake and standard deviation of total, classes and individual flavonoid by cognitive status in the MEAL cohort.

	Cognitive Status		
	Normal (n = 801)	Impaired (n = 82)	*P*-Value ^a^
	*Mean (SD)*		
Total flavonoids, mg/d	250.2 (170.1)	219.6 (238.2)	0.137
Flavan-3-ols, mg/d	86.10 (90.61)	76.13 (170.87)	0.394
Catechins, mg/d	51.82 (60.24)	49.69 (132.35)	0.793
Flavonols, mg/d	56.06 (40.33)	54.52 (61.39)	0.756
Quercetin, mg/d	0.77 (1.01)	0.70 (1.32)	0.543
Kaempferol, mg/d	0.25 (0.23)	0.20 (0.18)	0.053
Flavanones, mg/d	37.92 (40.12)	33.57 (51.39)	0.365
Hesperetin, mg/d	27.30 (28.94)	24.01 (36.11)	0.339
Naringenin, mg/d	6.43 (7.16)	4.82 (6.02)	0.051
Flavones, mg/d	7.86 (6.88)	8.77(10.66)	0.284
Apigenin, mg/d	0.008 (0.004)	0.009 (0.008)	0.362
Luteolin, mg/d	3.98 (3.72)	4.38 (4.26)	0.360
Anthocyanins, mg/d	57.03 (54.79)	42.14 (44.18)	0.017

^a^ Differences were assessed by ANOVA test.

**Table 3:** Odds ratios (ORs) and 95% confidence intervals (CIs) for the association between flavonoid intake (total, classes and individual compounds) and impaired cognitive status in the MEAL cohort.

	Flavonoid Quartiles, OR (95% CI)			
	Q1 (n = 187)	Q2 (n = 243)	Q3 (n = 255)	Q4 (n = 198)
*Total flavonoids*				
Energy-adjusted ^a^	1	0.35 (0.19, 0.65)	0.15 (0.07, 0.34)	0.48 (0.24, 0.94)
Multivariate ^b^	1	0.37 (0.17, 0.79)	0.13 (0.05, 0.35)	0.39 (0.15, 1.00)
*Flavan-3-ols*				
Energy-adjusted ^a^	1	1.71 (0.75, 3.88)	1.25 (0.42, 3.73)	1.29 (0.28, 5.85)
Multivariate ^b^	1	0.57 (0.26, 1.25)	0.30 (0.11, 0.76)	0.66 (0.29, 1.48)
*Catechins*				
Energy-adjusted ^a^	1	0.68 (0.38, 1.21)	0.53 (0.29, 0.99)	0.28 (0.11, 0.65)
Multivariate ^b^	1	0.56 (0.27, 1.18)	0.45 (0.20, 1.04)	0.24 (0.08, 0.72)
*Flavonols*				
Energy-adjusted ^a^	1	0.90 (0.50, 1.59)	0.36 (0.17, 0.75)	0.75 (0.38, 1.47)
Multivariate ^b^	1	0.57 (0.26, 1.25)	0.30 (0.11, 0.76)	0.66 (0.29, 1.48)
*Quercetin*				
Energy-adjusted^a^	1	1.49 (0.80, 2.79)	1.30 (0.70, 2.42)	0.63 (0.29, 1.36)
Multivariate^b^	1	0.81 (0.33, 2.00)	1.29 (0.57, 2.90)	0.30 (0.10, 0.91)
*Kaempferol*				
Energy-adjusted ^a^	1	0.90 (0.47, 1.69)	0.53 (0.27, 1.05)	0.80 (0.41, 1.57)
Multivariate ^b^	1	0.56 (0.25, 1.24)	0.45 (0.20, 1.04)	0.46 (0.16, 1.34)
*Flavanones*				
Energy-adjusted ^a^	1	0.77 (0.42, 1.43)	0.73 (0.39, 1.37)	0.56 (0.27, 1.15)
Multivariate ^b^	1	0.92 (0.41, 2.08)	0.88 (0.39, 1.97)	1.05 (0.43, 2.55)
*Hesperetin*				
Energy-adjusted ^a^	1	0.80 (0.43, 1.47)	0.75 (0.40, 1.40)	0.57 (0.27, 1.16)
Multivariate ^b^	1	1.08 (0.48, 2.40)	0.92 (0.41, 2.05)	1.20 (0.49, 2.92)
*Naringenin*				
Energy-adjusted ^a^	1	0.76 (0.41, 1.42)	0.62 (0.31, 1.22)	0.58 (0.28, 1.20)
Multivariate ^b^	1	0.69 (0.31, 1.56)	0.56 (0.24, 1.33)	1.00 (0.41, 2.39)
*Flavones*				
Energy-adjusted ^a^	1	0.90 (0.47, 1.74)	1.04 (0.54, 1.99)	1.27 (0.66, 2.45)
Multivariate ^b^	1	0.76 (0.34, 1.71)	1.09 (0.49, 2.44)	0.73 (0.31, 1.69)
*Apigenin*				
Energy-adjusted ^a^	1	0.69 (0.35, 1.37)	0.60 (0.30, 1.18)	0.89 (0.48, 1.63)
Multivariate ^b^	1	1.02 (0.45, 2.35)	0.41 (0.17, 1.02)	0.92 (0.40, 2.12)
*Luteolin*				
Energy-adjusted ^a^	1	0.56 (0.27, 1.16)	1.04 (0.55, 1.99)	1.41 (0.74, 2.69)
Multivariate ^b^	1	0.28 (0.10, 0.75)	0.93 (0.43, 2.01)	0.90 (0.40, 2.00)
*Anthocyanins*				
Energy-adjusted ^a^	1	0.80 (0.44, 1,46)	0.54 (0.28, 1.04)	0.65 (0.32, 1.31)
Multivariate ^b^	1	0.88 (0.40, 1.93)	0.49 (0.21, 1.15)	0.38 (0.14, 1.00)

^a^ Multivariate logistic regression adjusted for total energy intake (continuous). ^b^ Multivariate logistic regression adjusted for age (continuous), sex (male/female), total energy intake (continuous), body mass index (continuous), physical activity (low/medium/high), educational status (low/medium/high), occupational status (unemployed/low/medium/high), smoking status (current/former/never), alcohol consumption (no/moderate/regular), occurrence of hypertension, diabetes, dyslipidaemias, cardiovascular disease, cancer (yes/no), menopausal status (women only, yes/no).
